# Dual Mode Antibacterial Activity of Ion Substituted Calcium Phosphate Nanocarriers for Bone Infections

**DOI:** 10.3389/fbioe.2015.00059

**Published:** 2015-05-01

**Authors:** T. S. Sampath Kumar, K. Madhumathi, Y. Rubaiya, Mukesh Doble

**Affiliations:** ^1^Medical Materials Laboratory, Department of Metallurgical and Materials Engineering, Indian Institute of Technology Madras, Chennai, India; ^2^Department of Biotechnology, Indian Institute of Technology Madras, Chennai, India

**Keywords:** doxycycline, calcium phosphate bioceramics, antibacterial ion substitutions, bone infections, silver, zinc, strontium, calcium deficient hydroxyapatite

## Abstract

Nanotechnology has tremendous potential for the management of infectious diseases caused by multi-drug resistant bacteria, through the development of newer antibacterial materials and efficient modes of antibiotic delivery. Calcium phosphate (CaP) bioceramics are commonly used as bone substitutes due to their similarity to bone mineral and are widely researched upon for the treatment of bone infections associated with bone loss. CaPs can be used as local antibiotic delivery agents for bone infections and can be substituted with antibacterial ions in their crystal structure to have a wide spectrum, sustained antibacterial activity even against drug resistant bacteria. In the present work, a dual mode antibiotic delivery system with antibacterial ion substituted calcium deficient hydroxyapatite (CDHA) nanoparticles has been developed. Antibacterial ions such as zinc, silver, and strontium have been incorporated into CDHA at concentrations of 6, 0.25–0.75, and 2.5–7.5 at. %, respectively. The samples were found to be phase pure, acicular nanoparticles of length 40–50 nm and width 5–6 nm approximately. The loading and release profile of doxycycline, a commonly used antibiotic, was studied from the nanocarriers. The drug release was studied for 5 days and the release profile was influenced by the ion concentrations. The release of antibacterial ions was studied over a period of 21 days. The ion substituted CDHA samples were tested for antibacterial efficacy on *Staphylococcus aureus* and *Escherichia coli* by MIC/MBC studies and time-kill assay. AgCDHA and ZnCDHA showed high antibacterial activity against both bacteria, while SrCDHA was weakly active against *S. aureus*. Present study shows that the antibiotic release can provide the initial high antibacterial activity, and the sustained ion release can provide a long-term antibacterial activity. Such dual mode antibiotic and antibacterial ion release offers an efficient and potent way to treat an incumbent drug resistant infection.

## Introduction

Bone infections are becoming a frequent occurrence due to human longevity, increasing usage of prosthetic implants, and prevalence of drug resistant bacteria. These antibiotic resistant infections place immense burden on the health care system due to the medical complications, long duration of hospitalization, and associated costs. Bone and joint infections mostly fall into three types namely, osteomyelitis (infection of bones), septic arthritis (infection of joints), and prosthetic joint infections (Bejon and Robinson, 2013). Gram-positive bacteria like *Staphylococcus aureus*, β*-hemolytic Streptococci, Enterococci, Coagulase-negative Staphylococci, Haemophilus influenza, Escherichia coli*, and *Enterobacteriaceae* are commonly implicated in these infections (Bejon and Robinson, 2013). Osteomyelitis is caused by bacteria introduced through trauma, surgery, direct colonization from a proximal infection, or through systemic circulation. Prosthetic joint infections are most often hospital acquired or nosocomial in origin. Contamination of implant site can occur from sources like the air of the operating room and resident bacteria on the patient’s skin and body. Bacterial adhesion on the prosthesis results in biofilm formation, which renders bacteria resistant to most first-line antibiotics (Gristina, 1987). It is estimated that every year more than 2.2 million people are treated surgically for musculoskeletal disorders (Gentleman and Polak, 2006). Another study indicates the incidence of bone infections as approximately 5% of surgeries for fracture fixation devices, 2% of primary joint replacements, and 1.4–4% of total hip and knee replacements (Verron et al., 2012).

Treatment of bone infection involves systemic (intravenous) antibiotic administration in acute conditions and surgical excision of necrosed bone in chronic infections. Systemic administration of antibiotics suffers from side effects and is influenced by factors like the dosage interval and duration (Wu and Grainger, 2006). Bone infections associated with poorly vascularized or necrotic areas receive fluctuating and inadequate dose not capable of destroying the bacterial biofilm. Due to these problems, local drug delivery of antibiotics is favored. The advantages of local delivery include lowered dose, greater control over drug bioavailability and release profile, maintenance of therapeutic concentration at the infection site, avoidance of side effects, and lowered cost (Huh and Kwon, 2011). Currently, the gold standard in local delivery for bone infections is poly methyl methacrylate (PMMA) containing gentamycin available as beads and cement (McLaren, 2004). Various other biopolymers have been tried as drug carriers. However, most polymers are not bioactive, mostly non-biodegradable, and the degradation products produce inflammatory reactions at the local site (Madhumathi and Sampath Kumar, 2014). Bone destruction and defects commonly occur because of bone infections or post surgery. In such cases, osteoconductive or osteoinductive biomaterials promoting bone repair, and regeneration is preferable over polymers as delivery vehicles of antibiotics.

Bioactive ceramics such as calcium phosphate ceramics (CPCs) are attractive candidates for local drug delivery. Hydroxyapatite (HA), calcium deficient hydroxyapatite (CDHA), and tricalcium phosphate (TCP) are some of the CPCs of interest in bone therapeutics. Their degradation products are calcium and phosphate ions, which are commonly present in the human body (Bose and Tarafder, 2012). Human bone is considered as a composite of collagen biopolymer (~20%), reinforced with carbonated non-stoichiometric apatite (~70%) nanocrystals (Dorozhkin, 2010). These apatite nanocrystals have plate like morphology with width 15–30 nm and length 30–50 nm. Among the CPCs, CDHA [Ca_10-x_(HPO_4_)_x_(PO_4_)_6-x_(OH)_2-x_; Ca/P = 1.33–1.66] with tailorable Ca/P ratio and degradability is preferred for drug delivery applications (Victor and Kumar, 2008; Madhumathi and Sampath Kumar, 2014). CDHA is structurally similar to stoichiometric HA (non-biodegradable) and compositionally can be varied even to that of TCP (rapidly biodegradable) (Victor and Kumar, 2008). The apatitic structure presents multiple functional groups to which many biomolecules can bind. Our earlier studies have shown that CDHA nanoparticles of Ca/P ratio 1.61 exhibit the maximum uptake and release of drugs like doxycycline and tetracycline (Victor and Kumar, 2008; Madhumathi and Sampath Kumar, 2014).

**Figure 1:**
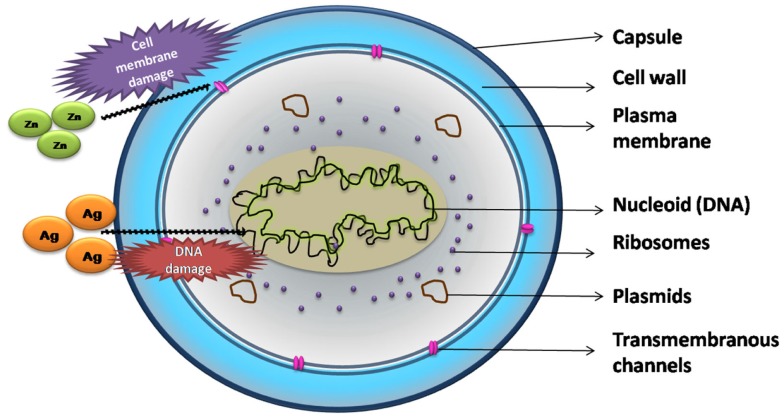
**Major mechanism of action of ions on *Staphylococcus aureus***.

Although local drug delivery has more advantages compared to systemic delivery, the main challenge lies in treating drug resistant infections. Drug resistance results in reduced efficacy of antibacterial drugs, increasing the morbidity and mortality. The 2014 WHO global report on drug resistance projects an alarming scenario where common infections and minor injuries can kill humans in the near future (World Health Organization, 2014). Some alternate approaches explored include stimulation of the body’s natural defense system by delivering biological molecules like cytokines that stimulate cell-mediated immunity (Li et al., 2009; Boyce et al., 2012), local delivery of antimicrobial peptides (Costa et al., 2011) etc., and developing antimicrobial materials such as antimicrobial polymers (Siedenbiedel and Tiller, 2012), antibacterial nanoparticles (Madhumathi et al., 2010), and a combination of these approaches (e.g., antibiotics with metallic nanoparticles) (Gu et al., 2003) to which drug resistant pathogens are susceptible. Ions such as silver, zinc, and strontium exhibit antibacterial activity against many bacteria. Their mode of action against bacteria is shown in Figure 1. Silver exerts its antibacterial activity by both deactivating the mitochondrial enzymes as well as denaturing the DNA of the bacterium (Rameshbabu et al., 2007). Strontium ions, in addition to being antibacterial, are known to up regulate osteoblast proliferation and down regulate osteoclast formation. The bacteriostatic activity of strontium substituted CDHA has been related to its highly negative zeta potential compared to pure CDHA (Ravi et al., 2012). Zinc is not only antibacterial, it also promotes the proliferation of osteoblasts while inhibiting osteoclastic bone resorption. In addition, it also exhibits anti-inflammatory properties. Zinc ions inhibit bacterial growth by binding to the bacterial membrane affecting calcium uptake and changing the membrane fluidity (Venkatasubbu et al., 2011). Some of the ions can also be toxic to mammalian cells. Hence, the above antimicrobial ions incorporated CPCs have been studied for their antibacterial activity and biocompatibility to establish the therapeutic limit of ion substitution (Rameshbabu et al., 2007; Venkatasubbu et al., 2011; Ravi et al., 2012). The ion substituted bioceramics present salient features such as cost effective preparation, long-term storage, and are amenable to sterilization (Huh and Kwon, 2011). Using the ion substituted bioceramics as carrier for antibiotics offers multiple advantages such as rapid and sustained antibacterial effect, broad-spectrum activity, and in some cases increased potency of drugs (Venkatasubbu et al., 2011).

**Figure 2:**
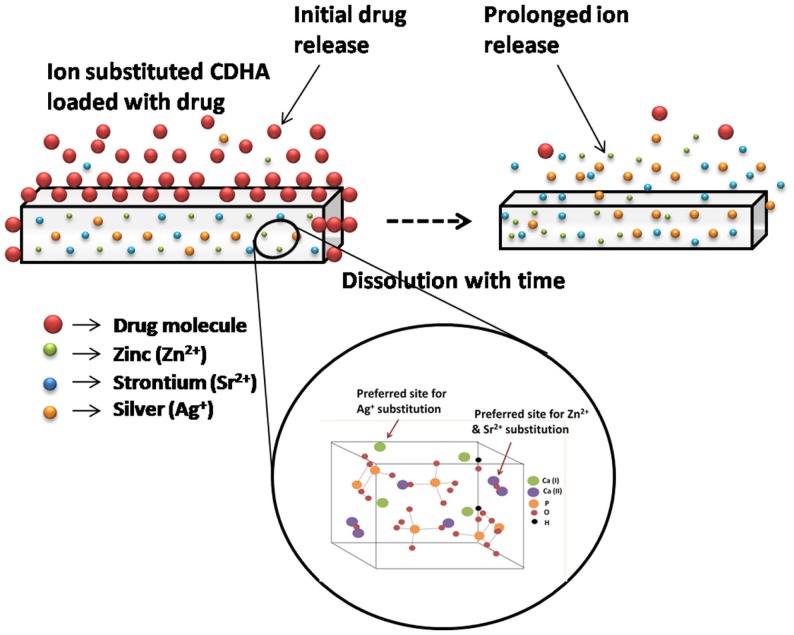
**Schematic illustration of dual mode CDHA nanocarriers with initial drug release followed by sustained ion release**.

In this work, an antibiotic delivery system based on antimicrobial ion substituted CDHA nanoparticles with dual mode activity as graphically represented in Figure 2 was developed. The antibacterial activity was provided by the antibiotic released during the initial phase, which is followed by that of the sustained ion release to contain infections. Ion substituted CDHA of Ca/P 1.61 was prepared by incorporating zinc (Zn^2+^), silver (Ag^+^), and strontium (Sr^2+^) ions separately at optimal concentrations for the antibacterial effect. All these ions are substituted at the calcium sites of apatite in exchange for calcium ions. While Ag^+^ is substituted for Ca^2+^ at Ca(I) cation site, the Zn^2+^ and Sr^2+^ ions can be substituted at the Ca(II) site. The zinc substitution was fixed at 6 at. % since, zinc substituted HA was also shown to exhibit beneficial anti-inflammatory activity at 5% concentration (Velard et al., 2010). Concentrations more than 6% were avoided due to concerns of toxicity. Silver substitution in CDHA was fixed at 0.25–0.75 at. % substitution based on earlier studies, where silver substituted HAs exhibited both antibacterial activity and biocompatibility at 0.5% substitution while 1% substitution showed toxicity (Rameshbabu et al., 2007). Another study has shown that CDHA was both antibacterial and biocompatible at 5 at. % strontium substitution (Ravi et al., 2012). Hence, the strontium substitution was varied between 2.5 and 7.5 at. %. Pure as well as ion substituted CDHAs were synthesized by a microwave accelerated wet chemical synthesis method, which is a rapid method to produce highly pure nanoparticles of narrow size distribution (Siddharthan et al., 2004). Doxycycline is a potent antibiotic, which also exhibits anti collagenase activity thereby preventing host mediated tissue destruction (Victor and Kumar, 2008). The effects of ion substitutions on the drug release, antibacterial activity, and biocompatibility of CDHA nanoparticles have been studied and analyzed.

## Materials and Methods

### Materials

Calcium nitrate [Ca_3_(NO_4_)_2_⋅4H_2_O], diammonium hydrogen phosphate [(NH_4_)_2_HPO_4_], zinc nitrate hexahydrate [Zn(NO_3_)_2._6H_2_O], strontium nitrate [Sr(NO_3_)_2_], and ammonia (30% GR) were purchased from MERCK, India. Silver nitrate (AgNO_3_) was purchased from SDFCL, India. All chemicals were pure and of analytical grade. Doxycycline hyclate was purchased from Sigma-Aldrich, India.

### Synthesis

Pure and ion substituted CDHAs were prepared as reported earlier (Rameshbabu et al., 2007; Ravi et al., 2012). CDHA nanoparticles were synthesized using a microwave accelerated wet chemical synthesis method using Ca_3_(NO_4_)_2_⋅4H_2_O and (NH_4_)_2_HPO_4_ as precursor solutions mixed at a Ca/P ratio of 1.61. The pH during the synthesis was maintained above 10 using ammonia. After complete mixing, the solution was subjected to irradiation in a microwave oven (BPL, India) of 800 W for about 30 min using 60% of the power. The precipitate was then washed thrice with distilled water to remove ions such as NH^4+^ and ${\text{NO}}_{3}^{2-}$, oven dried at 100°C, and ground to a fine powder using an agate mortar and pestle. The zinc, silver, and strontium substituted CDHAs were synthesized following the same procedure, with the addition of their respective solutions such as Zn(NO_3_)_2_⋅6H_2_O/AgNO_3_/Sr(NO_3_)_2_ to the precursor solution of Ca_3_(NO_4_)_2_⋅4H_2_O and titrated with (NH_4_)_2_HPO_4_ to obtain (Ca + X)/P ratio of 1.61 (where X = Zn or Ag or Sr). The ion substituted CDHAs with 6 at. % zinc, 0.25–0.75 at. % silver, and 2.5–7.5 at. % strontium were coded as listed in Table 1.

**Table 1:** **List of cell parameters, crystallite size, loading and release profiles for ion substituted CDHAs**.

Sample code	% ionic substitution	Cell parameters (Å)		Cell volume (Å)^3^	Average crystallite size (nm)		Doxycycline loading percentage (%) (mean ± SD)	Doxycycline release percentage (%) (mean ± SD)
		a	c		XRD	TEM (mean ± SD)	
CDHA	–	9.110	6.80	519	25	(41 ± 0.6) × (5 ± 0.1)	68 ± 9	61 ± 1.0
ZnCDHA	6	9.014	6.72	509	19	(39 ± 0.7) × (4 ± 0.3)	47 ± 8	57 ± 1.2
0.25AgCDHA	0.25	9.190	6.82	524	27	(41 ± 0.5) × 6 ± 0.3)	37 ± 4	49 ± 1.6
0.5AgCDHA	0.5	9.195	6.81	527	28	(42 ± 0.9) × (6 ± 0.4)	30 ± 3	52 ± 0.8
0.75AgCDHA	0.75	9.198	6.83	529	29	(44 ± 0.5) × (6 ± 0.4)	27 ± 5	55 ± 0.8
2.5SrCDHA	2.5	9.410	6.80	541	34	(47 ± 0.5) × (6 ± 0.3)	31 ± 6	51 ± 1.1
5SrCDHA	5	9.440	6.82	547	38	(49 ± 0.4) × (6 ± 0.2)	26 ± 5	54 ± 0.7
7.5SrCDHA	7.5	9.460	6.83	549	38	(51 ± 0.2) × (6 ± 0.2)	21 ± 6	56 ± 0.9

### Material characterization

The nanocarriers were characterized for phase purity and structural analysis by X-ray powder diffraction method (XRD, Bruker D8 DISCOVER, USA) using Cu Kα radiation (λ = 1.54 Å). The diffraction patterns were recorded with step size of 0.1°/step and at a scanning rate of 1 step/s. The functional groups present in pure CDHA and ion substituted CDHA nanocarriers were analyzed in the spectral range of 4000–510 cm^−1^ by Fourier transform infrared spectroscopy (Spectrum Two FT-IR spectrometer, Perkin-Elmer, USA) in the attenuated internal reflection (ATR) mode. Transmission electron microscopy was used to identify the morphology of the CDHA samples. The samples were dispersed in acetone and sonicated for 15 min using an ultrasonic bath (Citizen, India) at frequency of 45 kHz. The dispersions were dropped on carbon-coated copper grids, dried, and examined with a transmission electron microscope (Philips CM20 TEM, Netherlands) operated at 120 kV. The particle size analysis and zeta potential measurements of the pure and ion substituted CDHAs were carried out by dynamic light scattering (DLS) technique (Malvern Zetasizer Nano ZS-90, UK). One milligram of the samples were dispersed in 10 ml distilled water and sonicated for 15 min. One milliliter of the supernatant was then removed and used for DLS measurements.

### *In vitro* loading and release studies of doxycycline

Loading and release studies of doxycycline from pure CDHA and ion substituted CDHA were performed as described earlier (Madhumathi and Sampath Kumar, 2014). About 10 mg of the drug was dispersed in 10 ml of phosphate buffer solution (PBS) of pH 7.4. To this, 10 mg of CDHA nanocarriers was added. The samples were placed in water bath at 37°C for 24 h. After 24 h, 2 ml of supernatant was removed for estimation of doxycycline concentration at 274 nm using UV-Vis spectrophotometer (Lambda 35, Perkin-Elmer, USA). The samples were centrifuged and dried at room temperature for 24 h. The amount of drug loaded onto the nanocarriers was determined by the following equation:

(1) $$\%\text{Drug loading}={\text{I}}_{\text{c}}-{\text{F}}_{\text{c}}/{\text{I}}_{\text{c}}\times 100$$

where *I*_c_ and *F*_c_ are initial and final concentration of doxycycline in PBS. The release study was performed by dispersing 10 mg of doxycycline loaded CDHA nanocarriers in PBS solution of pH 7.4 kept in a constant temperature water bath at 37°C. About 2 ml of supernatant was removed for doxycycline estimation and replaced by fresh PBS at periodic intervals over a period of 7 days. The drug release profile was determined by measuring the absorbance values at different time intervals (*F*_c_) from the initial concentration (*I*_c_). All the experiments were performed in triplicates.

### *In vitro* dissolution and ion release studies

The *in vitro* dissolution studies were carried out in PBS of pH 7.4 kept in a constant temperature water bath and maintained at 37°C over a period of 21 days. Ten milligrams of the samples in powder form were dispersed in 10 ml of PBS at a concentration of 1 mg/ml. The weight loss of the samples, pH variations as well as the ionic concentration in the supernatant solution, was regularly monitored. At the end of each experiment, the samples were filtered, dried at 100°C, and weighed to calculate the percentage weight loss. The ion release studies were performed by removing 2 ml of the supernatant from each sample and diluting with 23 ml of distilled water to obtain a total volume of 25 ml. The concentration of the released Zn^2+^, Ag^+^, and Sr^2+^ ions were determined using an inductively coupled plasma optical emission spectrometer (ICP-OES) (PerkinElmer Optima 5300 DV, USA). These studies were carried out in triplicates for 1^st^, 3^rd^, 7^th^, 14^th^, and 21^st^ day.

### *In vitro* antibacterial studies

Various antibacterial studies such as minimum inhibitory concentration (MIC), minimum bactericidal concentration (MBC), time-kill assays were performed on ion substituted CDHAs against the *E. coli* (NCIM 2931) and *S. aureus* (NCIM 5021), which were purchased from the National Chemical Laboratory, Pune, India. The bacteria were stored in glycerol stock at -20°C and used after revival as and when required. The stage of bacteria was determined by plotting a growth curve. They were found to be in stationary phase for MIC/MBC and bacterial growth inhibition studies. However, they were in log phase for time kill assay in view of the longer duration of the bacterial study. Pure CDHA was used as the control. All tests were performed in triplicates.

#### Minimum Inhibitory Concentration

Minimum inhibitory concentration is the lowest concentration of an antimicrobial agent that inhibited the visible growth of microorganism after incubation of 24 h. Ion substituted CDHA nanoparticles were suspended in nutrient broth at concentrations of 300, 200, 100, 75, 50, 25, 10, and 5 mg/ml and ultrasonicated to ensure optimal dispersion. After 24 h, 10 μl of the inoculum of each microorganism was added to the nanoparticulate suspension. The suspension was then incubated at 37°C for 24 h in a shaking incubator (180 rpm). Nanoparticle-free broths containing bacterial inoculum were used as negative controls. Resazurin dye was used to assess the viability of bacteria with presence of blue color indicating bacterial growth inhibition and a change to red color indicating viable bacteria. About 10 μl of 0.01% resazurin solution was added to the suspension and incubated for 2 h. The MIC of the ion substituted CDHAs was calculated as the lowest concentration of the nanoparticles that did not permit any visible growth of bacteria (blue color) during 24 h of incubation.

#### Minimum Bactericidal Concentration

Minimum bactericidal concentration refers to the lowest concentration of an antibacterial agent required to kill the bacteria. In order to determine the MBC, the MIC samples prepared at various concentrations were plated in nutrient agar plates. The plates were incubated at 37°C for 24 h. The lowest concentration at which no bacterial colony was observed was taken as the MBC.

#### Time-Kill Curve

Pellets of pure and ion substituted CDHA samples were prepared by uniaxial compaction. About 300 mg of the nanoparticulate powder was weighed and compacted in a bench press at a force of 15 kN. The time-kill curve of these pellets was plotted by testing against *S. aureus* bacteria. The pellets were added to 2 ml of nutrient broth containing 1 × 10^8^ CFU/ml in a 24-well plate and incubated at 37°C for 1, 3, 5, and 7 days. Pure CDHA was used as positive control while the bacteria containing broth without nanoparticles were used as negative control. After each incubation period, 100 μl of the broth solution was collected from the 24-well plate and was serially diluted to calculate the number of surviving colonies. About 50 μl of the aliquot of the latter was then added onto a nutrient agar and incubated at 37°C for 1 day for colony formation. The colonies formed were examined and counted. The time-kill curve was plotted as bacterial colony reduction (log CFU/ml) with time.

#### Bacterial Growth Inhibition Study on Drug Loaded Samples

Drug loaded ion substituted samples (1 mg) were added to 9 ml of the nutrient broth. The suspensions were then inoculated with 1 ml of *S. aureus* bacterial cultures and were incubated at 37°C for 24 h with shaking. The antibacterial efficacy of the drug loaded ion substituted samples was determined from the optical density (OD) of the cultures at 600 nm. The antimicrobial reduction percentage was calculated using the following equation

(2) Bacterial reduction%={1−(sample OD/control OD)}×100%.

### *In vitro* biocompatibility studies

The biocompatibility of the drug loaded CDHA nanoparticles was tested against L6 myoblast cells (NCCS, Pune) by MTT [3-(4, 5-181dimethylthiazole-2-yl)-2, 5-diphenyl tetrazolium bromide] assay. MTT assay is a colorimetric test based on the selective ability of viable cells to reduce the tetrazolium component of MTT into purple colored formazan crystals. The L6 myoblast cells were grown to confluence with Dulbecco’s modifed eagle’s medium (DMEM), supplemented with 10% fetal bovine serum (FBS) and 1% 100× antibiotic-antimycotic liquid, and incubated at 37°C with 5% carbon dioxide in a CO_2_ incubator (Astec, Japan). The cells were then trypsinized and the number of cells was counted with the help of a hemocytometer (Marienfeld, Germany). They were then diluted (10^4^ cells per well) and seeded in 96-well plates and cultured for 24 h. One milligram of the CDHA samples was suspended in 1 ml of DMEM and incubated at 37°C for 24 h. The media in the 96-well plates were then replaced with 100 μl of the supernatant from the CDHA samples and again incubated for 24 h. About 20 μl of 5 mg/ml MTT was added to each well and incubated for 4 h. The formazan precipitates were solubilized in dimethyl sulfoxide (DMSO) and the absorbance was measured at 570 nm using a multimode plate reader (EnSpire, Perkin-Elmer, Singapore). The percentage of viable cells was calculated as the percentage relative to the control (standard polystyrene tissue culture plates) using the following equation

(3) %Cell viability=(mean OD/control OD)×100.

### Statistical analysis

The values are expressed as mean ± SD. Statistical analysis was performed using one and two way ANOVA wherever applicable. The *p*-value <0.05 was considered statistically significant.

## Results

### Material characterization

**Figure 3:**
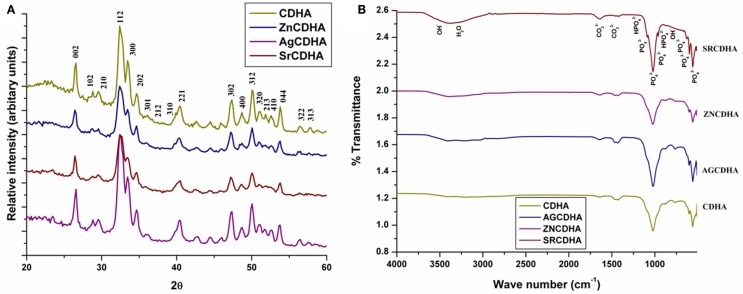
**Typical XRD spectra of CDHA and various ion substituted CDHAs (A)**. Typical FT-IR spectra of pure and ion substituted CDHAs **(B)**.

The XRD pattern of the CDHA and ion substituted CDHAs was compared with the standard HA pattern (JCPDS 09-432) as shown in Figure 3A. All the samples showed similar diffraction pattern corresponding to the characteristic peaks of HA. The absence of peaks other than HA confirms the formation of monophase CDHAs unaffected by ionic substitutions. The average crystallite size of the particles (*t*) was calculated from the broadening of the peak at 26° corresponding to (0 0 2) reflection using Scherrer’s formula [*t* = 0.9λ/Bcosθ], where λ = wavelength of CuKα radiation and B = full width at half maximum value (in radians) of the diffraction peak at 26°(2θ). The cell parameters and cell volume were calculated from the XRD data using the program “UnitCell” and are listed in Table 1, along with the crystallite size of the synthesized powders. It can be seen from Table 1 that the cell parameters varied with the type and concentration of substituted ions. Compared to pure CDHAs, the cell parameters and cell volume for zinc substituted CDHA showed a decrease while there was an increase in the case of silver and strontium substitution. The average crystallite size of pure CDHA was 25 nm. The crystallite size showed variations on ion substitution with values ranging from 19 nm for zinc to 27–32 nm in case of silver and strontium substituted CDHAs. The functional groups present in the ion substituted CDHAs were identified using FT-IR spectroscopy. Figure 3B shows the typical FT-IR spectra of pure and ions substituted CDHAs. All the characteristic vibration bands of CDHA such as ${\text{PO}}_{4}^{3-}$ (564, 603, 962, and 1032 cm^−1^), structural OH^−^ (633 and 3570 cm^−1^), and ${\text{CO}}_{3}^{2-}$ (1403 and 1455 cm^−1^) were present in all the samples (Siddharthan et al., 2004). The presence of ${\text{HPO}}_{4}^{2-}$ at 876 cm^−1^ confirms that the samples were CDHAs in nature. The absence of any other new bands suggests that the ions have been substituted into the crystal structure without the formation of any other intermediate compounds.

**Figure 4:**
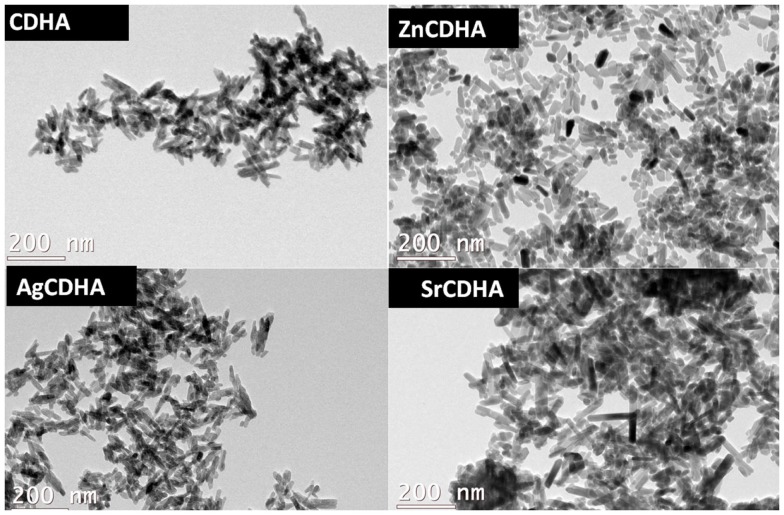
**Typical TEM images of CDHA and ion substituted CDHAs**.

The TEM images (Figure 4) show that the nanoparticles have a rod or acicular morphology. The particle sizes calculated from TEM micrographs using Image J software are listed in Table 1. Pure CDHA showed a size of (41 ± 0.6) × (5 ± 0.1) nm while the zinc substituted CDHA show a decrease in the size (39 ± 0.7) × (4 ± 0.3) nm. Both silver and strontium substituted CDHAs are larger compared to pure CDHA. In case of silver substitution, the size increases to a maximum of (44 ± 0.5) × (6 ± 0.4) nm approximately while strontium substituted CDHAs show an even larger size of (51 ± 0.2) × (6 ± 0.2) nm approximately. The particle size obtained from TEM correlates with that of XRD crystallite results.

### *In vitro* drug loading and release studies

**Figure 5:**
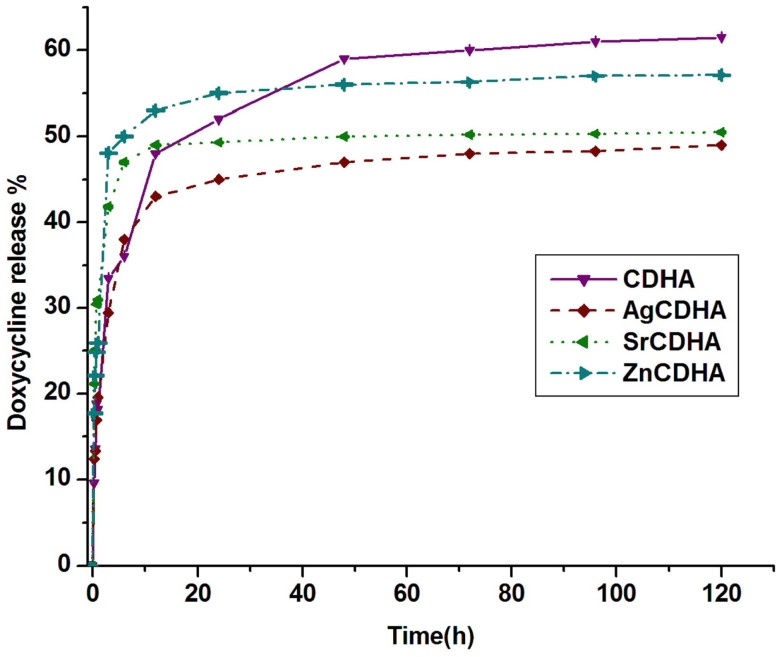
**Release profile of doxycycline from pure CDHA and ion substituted CDHAs (*n* = 3; data shown as mean ± SD; *p* < 0.05, one-way ANOVA)**.

The loading and release values of doxycycline from CDHA and ion substituted CDHAs are listed in Table 1. The loading and release percentage of doxycycline was found to be lower for all ion substituted samples compared to pure CDHA. In case of silver and strontium substituted samples, a decrease in loading of doxycycline with an increase in the amount of ion substitution was observed. Pure CDHA showed a doxycycline loading of 68 ± 9% and 69 ± 5% release. The zinc substituted CDHA showed a loading of 47 ± 8%, while silver substituted CDHAs showed between (27 ± 5) and (37 ± 4)%, and strontium substituted CDHAs showed a loading of (21 ± 6)–(31 ± 6)%. One-way ANOVA test was used to statistically analyze the samples in triplicates and the results were statistically significant with *p* < 0.05. The release percentage of doxycycline as listed in Table 1 indicates a different trend. A classic two-stage release with an initial burst release in 6 h followed by sustained release was observed. The release percentage of doxycycline has been found to decrease for all the substituted CDHAs. However, an increase in release was observed with increased ion substitution. The 6% zinc substituted CDHA, 0.25% silver substituted CDHA, and 2.5% strontium substituted CDHA samples with maximum loading and higher amount of total drug release were selected as the best samples for further particle size analysis, *in vitro* dissolution, and biological studies. The samples were coded as ZnCDHA, AgCDHA, and SrCDHA, respectively. The doxycycline loading percentage and release profile of these nanocarriers are shown in Table 1 and Figure 5.

### Particle size analysis and zeta potential measurements

The particle size analysis and zeta potential measurements of CDHA samples by DLS studies are listed in Table 2.

**Table 2:** **List of particle size and zeta potential of CDHA samples**.

Samples	Hydrodynamic diameter (d.nm) (mean ± SD)	Zeta potential (mV) (mean ± SD)
CDHA	1413 ± 16	−20.7 ± 0.4
AgCDHA	2181 ± 12	−14.9 ± 0.3
SrCDHA	1313 ± 07	−14.3 ± 1.0
ZnCDHA	2068 ± 21	−13.7 ± 0.3

The hydrodynamic diameter obtained is more than 1000 nm for all samples. The hydrodynamic diameter of AgCDHA and ZnCDHA was significantly higher than pure CDHA, while that of SrCDHA was the lowest. The negative surface charge of CDHA is reflected in the zeta potential values as expected. The ion substituted samples exhibit highly negative zeta potential compared to pure CDHA (Ravi et al., 2012). The studies were conducted as triplicates. The one-way ANOVA test results were statistically significant with *p* < 0.0001.

### *In vitro* dissolution and solubility studies

**Figure 6:**
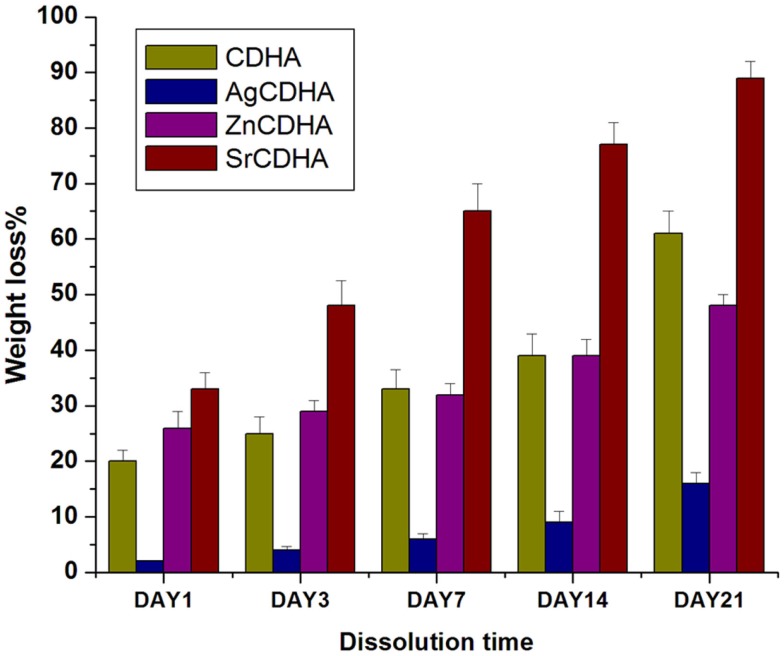
***In vitro* solubility studies showing the weight loss of ion substituted CDHAs in PBS of pH 7.4 at 37°C (*n* = 3; data shown as mean ± SD; *p* < 0.0001, two-way ANOVA)**.

**Figure 7:**
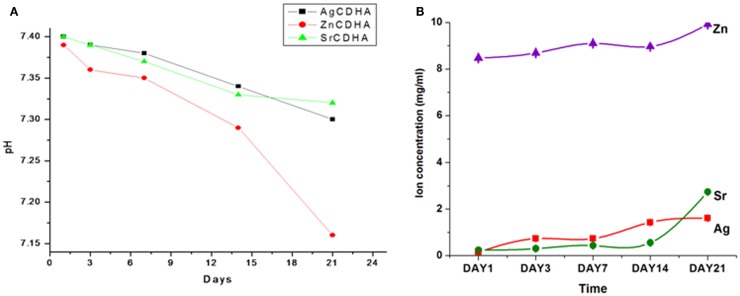
***In vitro* solubility studies showing pH fluctuations in PBS of pH 7.4 at 37°C (*n* = 3; data shown as mean; *p* < 0.005; one-way ANOVA) (A)**. *In vitro* dissolution studies of CDHA samples showing ion release in PBS of pH 7.4 at 37°C (*n* = 3; data shown as mean; *p* < 0.005, one-way ANOVA) **(B)**.

The *in vitro* dissolution of CDHA and ion substituted CDHAs were measured by the weight loss and pH variation as shown in Figures 6 and 7, respectively. All the synthesized nanopowders showed weight loss from the first day onward (Figure 6). Compared to pure CDHA, silver substituted CDHAs show a reduced weight loss suggesting lower solubility and higher stability. Strontium substitution on the other hand appears to increase the solubility of the CDHAs with more than 90% weight loss in a 21 day period. Although, the ZnCDHA exhibited a greater initial solubility than CDHA for up to 3 days, they show lower solubility than CDHA from seventh day onward and the difference becomes statistically significant by 21st day. The dissolution studies were analyzed by a two-way ANOVA test. The difference between the samples was found to be statistically significant with *p* < 0.0001. The pH values of the PBS solution in which ion substituted CDHAs were dispersed have also been found to decrease during this duration as shown in Figure 7A. There was a slight decrease in the pH from initial 7.4 to 7.32 for AgCDHA and SrCDHA and reaches 7.15 for ZnCDHA at the end of 21 days. The ion release profiles obtained from dissolution studies of the samples are shown in Figure 7B. Zinc ions were found at higher concentration followed by strontium and silver ions.

### *In vitro* antibacterial studies

The MIC and MBC values of the ion substituted samples are listed in Table 3. It can be seen that AgCDHA has the lowest MIC and MBC for both the bacteria even at low substitution. ZnCDHA showed a MIC of 200 μg/μl against both the bacteria. However, strontium substituted CDHAs showed very low antibacterial activity against *S. aureus* and no activity upto 300 μg/μl against *E. coli*. The MBC values also reflect a similar trend.

**Table 3:** **MIC and MBC values of various ion substituted CDHAs**.

Samples	*S. aureus*		*E. coli*	
	MIC (μg/μl)	MBC (μg/μl)	MIC (μg/μl)	MBC (μg/μl)
ZnCDHA	200	200	200	300
AgCDHA	20	25	10	20
SrCDHA	200	300	–	–

**Figure 8:**
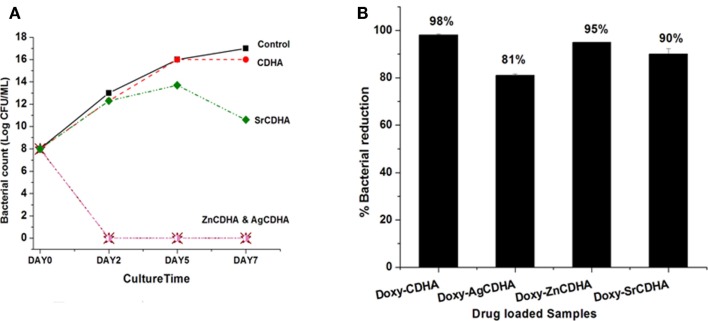
**Time-kill curve showing log reduction against *S. aureus* for ion substituted CDHA samples (A) (*n* = 3; data shown as mean; *p* < 0.0001, one-way ANOVA)**. Antibacterial activity of CDHA and ion substituted CDHA nanoparticles loaded with doxycycline (doxy – doxycycline) against *S. aureus* **(B)** (*n* = 3; data shown as mean ± SD; *p* < 0.005, one-way ANOVA).

The time-kill studies on *S. aureus* showed a reduction in bacterial growth for ion substituted samples. AgCDHA and ZnCDHA showed complete inhibition with absence of colonies on second day itself (Figure 8A). Two-way ANOVA was performed and a value of *p* < 0.0001 was obtained. The antibacterial activity of doxycycline loaded ion substituted CDHA nanoparticles were determined by incubating them with bacteria in a liquid broth. The results are shown in Figure 8B. It can be seen that there is a decrease in the antibacterial activity of the drug loaded substituted CDHAs compared to only drug loaded pure CDHA. Doxycycline loaded AgCDHA, especially, showed lower activity than other samples at the end of 24 h. The statistical analysis was calculated by one-way ANOVA with *p* < 0.05 considered significant.

### *In vitro* biocompatibility studies

**Figure 9:**
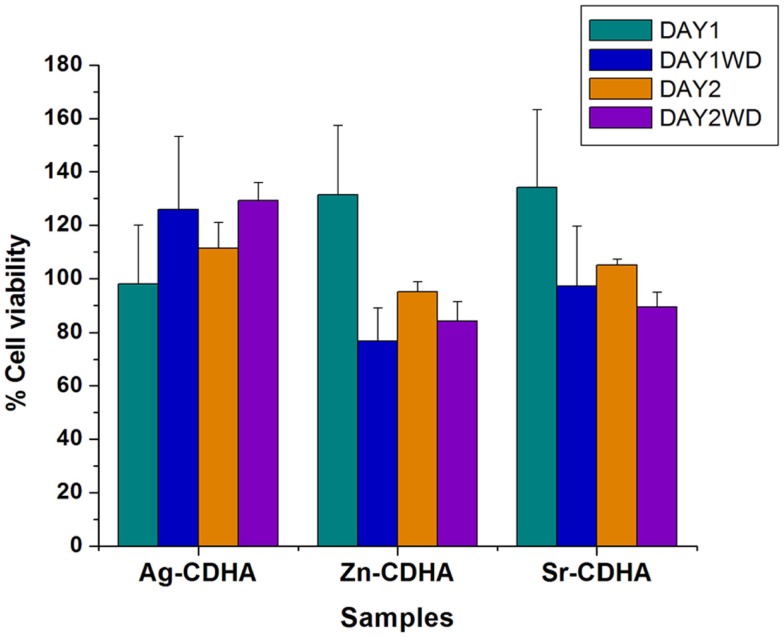
**Biocompatibility studies of pure and doxycycline loaded ion substituted CDHA samples (1 mg/ml) by MTT assay for 24 and 48 h (WD – with doxycycline) (*n* = 3; data shown as mean ± SD; *p* < 0.005, two-way ANOVA)**.

The biocompatibility of the samples before and after drug loading was tested by MTT assay and the results are shown in Figure 9. All samples were found to be biocompatible at a concentration of 1 mg/ml, with the cell viability above 80% at the end of 48 h. The biocompatibility of AgCDHA did not vary between 24 and 48 h of incubation while ZnCDHA and SrCDHA showed a decrease in cell viability after 24 h. Doxycycline loaded samples showed variations in biocompatibility in case of ZnCDHA and SrCDHA at the end of 24 h. Although, drug loaded AgCDHA did not show any statistical difference in cell viability, there was a reduction in cell viability for other drug loaded samples compared to pure samples at 24 h. At the end of 48 h, the cell viability of drug loaded samples showed no significant variation compared to that of 24 h incubation. The statistical analysis was conducted by two-way ANOVA test for triplicate samples with a significance of *p* < 0.005.

## Discussion

Conventional treatment of bone infections suffer from problems like emergence of drug resistance and persistence of infections due to inadequate dose, leading to poor healing of bone that can be addressed with the help of nanotechnology. A dual strategy of combining antibiotic release with antibacterial ion release can act effectively against persistent biofilms and can even prevent emergence of drug resistance. In this regard, CDHA nanoparticles offers a good platform for both ion substitution and drug release. The apatitic structure of CDHA provides multiple sites for ion substitution as well as drug/protein binding. CDHA also has higher bioactivity, higher specific surface area, and high efficient precipitation of bone like apatites compared to other calcium phosphates (Dorozhkin, 2010).

Highly pure ion substituted CDHA nanoparticles were obtained by microwave accelerated wet chemical method as evidenced from XRD, FT-IR, and TEM results. The ion substitutions have been found to influence structural parameters, drug loading and release capacity, particle stability, antibacterial activity, and biocompatibility. The cell volume, lattice parameters, and crystallite size vary with difference in the ionic radii of the substituting ions compared to calcium ions. ZnCDHA show a reduction in the cell parameters and size since Zn^2+^ ions have a smaller ionic radii of 0.74 Å compared to Ca^2+^ (0.99 Å). The above parameters have been found to increase in AgCDHAs and SrCDHAs, since Ag^+^ (1.28 Å) and Sr^2+^ (1.18 Å) ions have higher ionic radii than Ca^2+^.

The loading and release of doxycycline is also affected by the ion substitutions. Doxycycline binds to the Ca^2+^ site of CDHA (Victor and Kumar, 2008). A partial substitution of Ca^2+^ by other ions has reduced the binding sites for doxycycline as seen from the lower loading percentage. The loading decreases with increasing ion substitutions. It can be seen that even small amounts of silver substitution (<1at. %) show a high impact on doxycycline loading. The drug release percentage and profile was also affected by ion substitutions. The release profile shows the burst release of the drug from AgCDHA and SrCDHA, suggesting surface adsorption is the main mechanism of drug binding. It is surprising to observe that the biphasic release from ZnCDHA is closer to that of CDHA despite the relatively higher substitution. Since an optimal drug binding capacity is expected from the CDHA system, samples were selected based on the loading and release studies. Thus, 0.25% Ag^+^ substituted CDHA and 2.5% Sr^2+^ substituted CDHA was selected for further studies along with 6% Zn substituted CDHA.

*In vitro* dissolution and stability studies are essential to understand the behavior of ion substituted CDHA in physiological system. Since, the antibacterial activity is not only dependant on drug release but also on ion release, these studies assume significance. A 21 day study showed that silver substitution lowered the solubility while strontium increased the solubility of CDHA. Substitutions with zinc showed a lower solubility than pure CDHA at 21st day. At the end of 21 day period, SrCDHA sample showed the maximum solubility with more than 90% weight loss. Strontium substitutions are known to decrease the crystallinity of CDHA, which can be a factor for the high solubility (Aina et al., 2012; Ravi et al., 2012). The order of weight loss was SrCDHA > CDHA > ZnCDHA > AgCDHA. This also correlates well with the ion release, since zinc and strontium ion concentration in PBS was higher than silver ions during this period. The pH study was done in a buffer solution where immediate variation may not be visible, while ICP analysis indicates the actual ion concentrations at a higher resolution. Hence, the trend of pH values may not correlate with Zn ion concentrations at day 1. The stability of the nanoparticles in a liquid medium was also investigated. The particle size obtained by TEM and the hydrodynamic diameter obtained by DLS measurements do not correlate because of both anisotropic non-spherical nature as well as aggregation of the nanoparticles. Since, the particles are intended for local drug delivery, the aggregation of nanoparticles in aqueous medium should not pose any problem. The zeta potential values also reflect the unstable nature of the nanoparticles with values between -13.7 and -20.7 mV.

Antibacterial activity of ion substituted CDHAs before and after drug loading was evaluated by multiple studies including MIC/MBC determination and time-kill assay. The MIC and MBC studies test the bacteriostatic (growth inhibitory) and bactericidal (killing of bacteria) effects of the samples respectively. Of the three ions, silver is the most potent antibacterial agent, which is bactericidal in nature. AgCDHA is highly effective against both *S. aureus* and *E. coli*, as can be seen from the MIC and MBC values. ZnCDHA shows moderate antibacterial activity against both bacteria with similar MIC values. However, the MBC of zinc is higher for *E. coli*. This is because gram-positive bacteria like *S. aureus* are more susceptible to zinc ions than gram-negative *E. coli* due to the difference in the protein constituents of their cell walls (Jain et al., 2013). SrCDHA showed a weak antibacterial activity against *S. aureus*. However, no antimicrobial activity was observed against *E. coli* at upto 300 μg/μl. The time-kill assay was used to evaluate the time dependant antibacterial effect of ion substituted CDHAs on *S. aureus* bacterial growth. This assay was found to be ideal to evaluate the antibacterial action of ions released from substituted CDHAs in a time dependant manner. Similar studies for zinc substituted HA have been reported earlier (Thian et al., 2013). The trend observed in the MIC/MBC studies was also reflected in time-kill studies, with both AgCDHA and ZnCDHA inhibiting the bacterial growth, as their concentration (300 μg/μl) was more than their MBC. SrCDHA showed an incomplete growth reduction since its MBC was more than 300 μg/μl. The antibacterial activity of doxycycline loaded ion substituted samples against *S. aureus* present interesting results. There is an overall marginal reduction in the antibacterial activity of doxycycline loaded ion substituted CDHAs compared to pure CDHA at the end of 24 h. This can be explained in conjunction with the drug loading. The initial antibacterial activity is provided by the doxycycline drug, which is loaded at lower amounts in ion substituted samples. Silver shows the lowest loading (~31%) and hence its antibacterial activity is also lower than other samples at 24 h. Thus, though silver ion is highly antibacterial, it is released only by the time dependant dissolution of the CDHA nanoparticles during which the drug released provides the antibacterial activity.

Biocompatibility of ion substituted CDHAs is an important criteria for its clinical applications. Though the ions were substituted at concentrations deemed biocompatible, the biocompatibility of drug loaded substituted CDHAs had to be evaluated using MTT assay. The doxycycline concentration was approximately 0.18 μg/μl for CDHA, 0.26 μg/μl for ZnCDHA, 0.12 μg/μl for AgCDHA, and 0.15 μg/μl for SrCDHA samples as calculated from drug release studies at the end of 24 h. The doxycycline release reduces the cell viability during first 24 h for ZnCDHA and SrCDHA while no appreciable difference was observed for AgCDHA samples with and without drug loading. However, a statistically significant decrease in cell viability was observed for ZnCDHA and SrCDHA at the end of 48 h compared to 24 h. However, all the samples were found to be biocompatible at the given concentration. The results clearly demonstrate the advantage of ion substituted CDHAs, as the controlled release of ions incorporated into CDHAs plays an important role in improving the biocompatibility.

Among various ion substituted CDHAs, AgCDHA with 0.25% substitution shows the lowest doxycycline loading. However, it exhibits the highest antibacterial activity and is also biocompatible. The SrCDHA sample shows low loading of doxycycline and lowest antibacterial activity but exhibits higher biocompatibility. Although, the ZnCDHA sample exhibits reasonable drug loading and satisfactory antibacterial activity, it shows lower cell viability compared to pure CDHA and other ion substituted samples. The CDHA and AgCDHA samples exhibit drug release up to 5 days while there was no change in the drug release profile after first day for SrCDHA and ZnCDHA samples. But both ZnCDHA and AgCDHA show high antibacterial activity at day 2 in spite of their different drug release profiles. Although, SrCDHA show maximum drug release by first day, it exhibits antibacterial activity from day 2 onward. A long-term antibacterial and cytotoxic study may clearly bring out the combined role of ion substitution and drug release in combating antimicrobial resistance.

Thus, the present work demonstrates that by combining antibiotics and antimicrobial ions, a biocompatible, sustained, highly efficient antibacterial bioceramic system with additional beneficial properties such as anti-inflammatory activity or bone remodeling activity can be developed to efficiently contain and treat bone infections.

## Conflict of Interest Statement

The authors declare that the research was conducted in the absence of any commercial or financial relationships that could be construed as a potential conflict of interest.
